# Polyctenidae (Hemiptera: Cimicoidea) species in the Afrotropical region: Distribution, host specificity, and first insights to their molecular phylogeny

**DOI:** 10.1002/ece3.9357

**Published:** 2022-10-01

**Authors:** Tamara Szentiványi, Sándor Hornok, Áron B. Kovács, Nóra Takács, Miklós Gyuranecz, Wanda Markotter, Philippe Christe, Olivier Glaizot

**Affiliations:** ^1^ Department of Ecology and Evolution University of Lausanne Lausanne Switzerland; ^2^ Museum of Zoology Lausanne Switzerland; ^3^ Department of Parasitology and Zoology University of Veterinary Medicine Budapest Hungary; ^4^ ELKH‐ÁTE Climate Change: New Blood‐Sucking Parasites and Vector‐Borne Pathogens Research Group Budapest Hungary; ^5^ Veterinary Medical Research Institute Budapest Hungary; ^6^ Centre for Viral Zoonoses, Department of Medical Virology University of Pretoria Pretoria South Africa; ^7^ Centre for Ecological Research Vácrátót Hungary

**Keywords:** bat bug, Chiroptera, Cimicidae, distribution, ectoparasite, Polyctenidae, specificity

## Abstract

Polyctenidae bugs are rarely studied, hematophagous, and highly specialized ectoparasites of bats. There are only 32 described species worldwide, including six species in the Afrotropical region. Knowledge on these parasites is limited, and most studies are restricted to the New World polyctenid species. Here we report additional records of *Adroctenes horvathi* from Kenya and South Africa, as well as *Hypoctenes faini* from Rwanda. We present an updated list of published polyctenid records in the Afrotropical region indicating their host specificity and their geographical distribution. We report global infection patterns and sex ratio of polyctenids based on previously published data, including Old and New World species. Lastly, we demonstrate the first molecular phylogeny of Polyctenidae, showing their phylogenetic relationship with the closely related family Cimicidae.

## Graphical abstract

Polyctenidae bugs are very rare, hematophagous, highly specialized ectoparasites of bats. In this study, we demonstrate the first molecular phylogeny of the family Polyctenidae, showing their phylogenetic relationship with the closely related family Cimicidae. Additionally, we report global infection patterns and sex ratio of polyctenids based on previously published data, including Old and New World species.

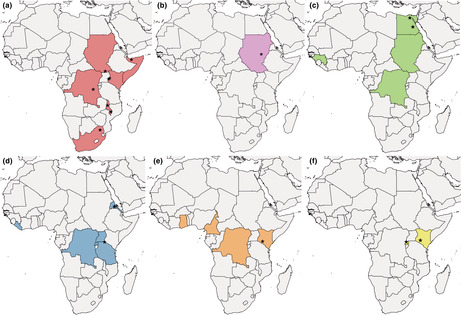

## 1 INTRODUCTION

### 1.1 Polyctenid diversity worldwide

Bats host a wide variety of parasites, including ectoparasitic bugs. Bat bugs (Hemiptera: Cimicidae and Polyctenidae) are blood‐sucking parasites, belonging to the superfamily Cimicoidea. Cimicids (especially the bed bugs, *Cimex lectularius* and *C. hemipterus*) are a well‐studied parasitic group as they are a public health concern due to their vectorial potential of several diseases, including *Trypanosoma cruzi* toward humans (Delaunay et al., 2011; Salazar et al., 2015). Additionally, the ecology, distribution, and phylogeny of some cimicids species parasitizing bats, particularly *C. adjunctus*, *C. pipistrelli*, *C. lectularius*, and closely related species, are relatively well studied (Balvín et al., 2014, 2013; Bartonička, 2008, 2010; Hornok et al., 2018, 2021, 2017; Quetglas et al., 2012; Reinhardt et al., 2007, 2008). By contrast, Polyctenidae is an extremely understudied ectoparasitic family. They are represented by 32 species worldwide belonging to two subfamilies and five genera (*Adroctenes* Jordan, 1912, *Eoctenes* Kirkaldy, 1906, *Hypoctenes* Jordan, 1922, *Polyctenes* Giglioli, 1864 within the Polycteninae and *Hesperoctenes* Kirkaldy, 1906 within the Hesperocteninae).

Polyctenid subfamilies occur in different biogeographical regions. Subfamily Polycteninae only found in the Eastern Hemisphere (Africa, Asia and Australia), whereas Hesperocteninae is restricted to the Western Hemisphere (South and North America) (Dick & Bindokas, 2007; Maa, 1964). In the Eastern Hemisphere, there are 16 species in total, out of which six species occur in the African continent. In the subfamily Polycteninae, *Eoctenes* is the most species rich genus with seven species [*E. coleurae* Maa, 1964, *E. ferrisi* Maa, 1964, *E. intermedius* (Speiser, 1904), *E. maai* Bhat, Sreenivasan and Ilkal, 1973, *E. nycteridis* (Maa, 1964) and references therein), *E. sinae* Maa (1961) and *E. spasmae* (Waterhouse, 1879)]. *Eoctenes intermedius* is the most widespread species with several records from Australia, Africa, and Asia (e.g. Malaya, Philippines, Sudan, Sumatra and Thailand) (Dick & Bindokas, 2007). By contrast, *Eoctenes coleurae* and *E. nycteridis* are endemic to the African continent. Additionally, three endemic species are found in the African region which are *Adroctenes horvathi*, *Hypoctenes clarus*, and *H. faini*. The most recent records of polyctenids from the African region indicate the occurrence of *Hypoctenes clarus* from Kenya, which was also a new observation to the country (Patterson et al., 2018). Nevertheless, the last polyctenid records were published nearly two decades ago from the continent (Kock et al., 1998), which suggests either biased sampling efforts, the difficulty of collecting polyctenids, or possibly the rarity of these parasites.

### 1.2 Phylogenetic relation with Cimicidae

The phylogenetic relationship of polyctenids with other groups has previously received little attention. It has been shown that, based on morphological characters, the phylogenetic relationship between cimicids and polyctenids represents two different monophyletic groups, but molecular data were missing from polyctenids (Schuh et al., 2009). Polyctenids are generally excluded from molecular phylogenetic reconstruction of the superfamily Cimicoidea, due to the lack of available specimens and molecular data on these species (Jung & Lee, 2012; Roth et al., 2019; Schuh et al., 2009). Only a cytochrome c oxidase subunit 1 mitochondrial gene (COI) fragment of a North American species, *Hesperoctenes fumarius*, has been previously published (Smit & Miller, 2019). Additionally, fossil records of polyctenids are not available.

### 1.3 Reproduction biology of polyctenids

Our knowledge about the basic biology and ecology of these bat bugs is currently based on some long‐standing observational work, based on a few common species. The whole life cycle of polyctenids takes place on their hosts (Jordan, 1911; Marshall, 1982a), in contrast with cimicids, which only feed on the host but lay eggs on a substrate, such as the host's roost wall. Polyctenids show strong morphological and physiological adaptation to their parasitic lifestyle; they are viviparous, dorsoventrally flattened, eyeless, and wingless, and these features might strongly affect their host specificity and abundance through limited dispersal ability.

### 1.4 Host specificity and infection patterns

Previously published data have suggested that polyctenids show a high specificity to their bat hosts. Most species are described as oioxenous (i.e., specific to one certain host species) and/or stenoxenous (i.e., occurring on two or more congeneric host species) (Maa, 1964; Marshall, 1982a). An experimental study has shown that *Hesperoctenes fumarius*, a New World species, is able to survive and actively feed on different host species, when dispersal barriers are removed (Dick et al., 2009), although congeneric host species were used during this experiment. Overall, specificity and host preferences of polyctenid species are mostly unknown.

Limited data are available about the infection patterns, such as prevalence and abundance of polyctenid species on their hosts. *Hesperoctenes fumarius* showed prevalence of 21% on *Molossus rufus* as well as intensity of infestation (mean number of bat bugs on infected hosts) of 2.22 ± 2.86 (Esbérard et al., 2005). Presley (2011) also reported the infection patterns of *H. fumarius* on two hosts. The prevalence of *H. fumarius* was 26.8% and 13% on *Molossus molossus* and *M. rufus*, respectively. Additionally, he observed mean abundance (mean number of bat bugs per host) of 0.5 ± 1.14 and 0.4 ± 1.49 as well as mean intensity of 2.0 ± 1.43 and 3.2 ± 3.00 on *M. molossus* and *M. rufus*, respectively (Presley, 2011). *Hesperoctenes* species tend to show sex‐biased parasitism toward female bat hosts and in some cases, their abundance is affected by host morphological characters, such as body mass and/or forearm length, which may indicate the body condition of their hosts (Presley & Willig, 2008). Data on the sex ratio of polyctenids are scarce. Some studies reported mostly female biased sex ratio in adults, although sex ratio at emergence was unknown (Maa, 1964; Marshall, 1981, 1982a).

Our aim was to describe the specificity, sex ratio, and distributional patterns of polyctenids using published and field collected data along with specimens retrieved from museum collection, extending the current knowledge on the Polyctenidae family. Furthermore, we aimed to gain insights to the phylogenetic relationship of this family in relation to the closely related family Cimicidae, for the first time.

## 2 MATERIAL AND METHODS

### 2.1 Sampling and species identification

Opportunistic ectoparasite sampling was carried out by the Centre for Viral Zoonoses at University of Pretoria at several sites in South Africa, Rwanda, and Botswana. This was part of bio surveillance in both frugivorous and insectivorous bat species between 2008 and 2017. Bat species were identified based on morphological characters (Meester, 1986; Van Cakenberghe et al., 2017). Currently valid bat names are used throughout this work, whenever possible, based on batnames.org (Simmons & Cirranello, 2022). Parasites were individually placed into 70% ethanol. Voucher specimens are deposited at Museum of Zoology, Lausanne, Switzerland. Additionally, further polyctenid specimens were examined at the collection of California Academy of Sciences in San Francisco, CA (USA), and previously unpublished data were also added to this work. Morphological identifications were performed using Maa (1964) and Greenwood (1991).

### 2.2 DNA extraction and molecular analyses

Polyctenid samples were extracted non‐invasively (whole body), keeping whole specimens from external damage. Specimens were placed in separate tubes at 56°C for overnight digestion, using 20 μl Proteinase‐K and 180 μl ATL buffer (per sample) (Qiagen). DNA was extracted using DNeasy Blood and Tissue Kits (Qiagen) based on the protocol provided by the manufacturer. We targeted the COI gene (658 bp long fragment) for the molecular analysis, and we used the following primers: Lep1F (5′‐ATT CAA CCA ATC ATA AAG ATA TTG G‐3′), Lep1Fdeg (5′‐ATT CAA CCA ATC ATA AAG ATA TNG G‐3′), and Lep3R (5′‐TAT ACT TCA GGG TGT CCG AAA AAT CA‐3′) (Balvín et al., 2015). Polymerase chain reaction (PCR) master mix was prepared based on previously published protocol (Hornok et al., 2017). During amplification, the following steps were used: 1 cycle of 95°C for 5 min, 40 cycles of 94°C for 40 s, 53°C for 1 min, and 72°C for 1 min. Final extension of 1 cycle of 72°C for 10 min (Veriti 96‐Well Thermal Cycler, Applied Biosystems). Additionally, we targeted the 16S gene fragment (381–384 bp), with the primers 16S LR‐J (5′‐TTA CGC TGT TAT CCC TAA‐3′) and 16S LR‐N (5′‐CGC CTG TTT ATC AAA AAC AT‐3′) (Kambhampati & Smith, 1995; Simon et al., 1994). Fragments were amplified using PCR premix (AccuPower PCR PreMix, BIONEER) under the following conditions: 1 cycle of 95°C for 5 min, 35 cycle of 95°C for 30 s, 48°C for 30 s, and 72°C for 30 s. Final extension of 1 cycle of 72°C during 5 min (Veriti 96‐Well Thermal Cycler, Applied Biosystems). Furthermore, we targeted the 18S gene fragment (1200 and 800 bp long fragments), using primer pairs 18S‐1 (5′‐CTG GTT GAT CCT GCC AGT AGT‐3′) and 18S‐3 (5′‐GGT TAG AAC TAG GGC GGT ATC T‐3′), and 18S‐2 (5′‐AGA TAC CGC CCT AGT TCT AAC C‐3′) and 18S‐4 (5′‐GAT CCT TCT GCA GGT TCA CC‐3′) (Tian et al., 2008); however, only the shorter region (800 bp) was successfully retrieved. Lastly, the 28S rRNA gene fragment was also targeted using the primers 1274 (5′‐GAC CCG TCT TGA AAC ACG GA‐3′) and 1275 (5′‐TCG GAA GGA ACC AGC TAC TA‐3′) (Markmann & Tautz, 2005). Another PCR was also used targeting an approx. 700‐bp‐long part of the 28S rRNA gene, with the primers 28S‐FF (5′‐TTA CAC ACT CCT TAG CGG AT‐3′) and 28S‐DD (5′‐GGG ACC CGT CTT GAA ACA C‐3′) (Hillis & Dixon, 1991). However, the amplification and sequencing of the 28S rRNA gene of *Hypoctenes faini* were not successful with two different primer sets. PCR reactions of 18S and 28S amplifications were performed as reported (Hornok et al., 2021).

PCR products were visualized on 1.5% agarose gel. Biomi Ltd. and Microsynth AG performed purification and high‐throughput Sanger sequencing of the PCR products.

Sequences (in the following order: 16S rRNA, COI, and 18S rRNA) were concatenated in the Geneious Prime 2019.2.3 (Kearse et al., 2012) software. The alignment of the concatenated sequences was done with MAFFT algorithm (Katoh et al., 2002). The best fitting evolutionary model was selected as general time reversible (GTR) + G + I model by MEGA 11.0.10 (Kumar et al., 2018; Tamura et al., 2021), as it takes into account most parameters. A Bayesian consensus tree was created using the MrBayes (Huelsenbeck & Ronquist, 2001; Ronquist & Huelsenbeck, 2003) Geneious plugin, with GTR model with gamma distribution and invariant sites (GTR + G + I). The stationarity of posterior distribution was also examined using the Geneious plugin. The chain length was set to 5,000,000, sampling frequency to 500 and burn‐in length to 100,000. The gene partitions were treated as unlinked. The random seed was set to 21,231. The analysis of the Bayesian tree was done with the MEGA11 11.0.10 (Kumar et al., 2018; Tamura et al., 2021) software. Distribution maps of parasites were produced by using QGIS version 2.16.2.

References sequences of *A. horvathi* and *H. faini* can be obtained in GenBank under accession numbers: ON157489–ON182061.

## 3 RESULTS

### 3.1 Polyctenidae collected during this study

Three polyctenids (2 female adults and 1 nymph) were found belonging to two species: *Adroctenes horvathi* (*n* = 1, female) and *Hypoctenes faini* (*n* = 2, female and nymph), from one female of *Rhinolophus simulator* (in South Africa, 26. 09. 2017) and one female of *Otomops martiensseni* (Rwanda, 13. 12. 2008), respectively.

Five specimens of previously unidentified and unpublished polyctenids were recorded, representing *Adroctenes horvathi* in the collection of the California Academy of Sciences in San Francisco, CA (USA). The specimens were collected by James D. Hawkins (1 female, 25. 02. 1971, Busia District, N Mambale, Kenya; 2 females, 1 male, 1 nymph, 11. 03. 1971) from *Rhinolophus* spp. We included these records in Table 1.

**TABLE 1:** Published records of Polyctenidae from the African continent along with our field and museum collected data

Polyctenid species	Host species (current/valid name)	Host family	Host habitat type	Country	Location	References
*Adroctenes horvathi* Jordan (1912)	*Rhinolophus blasii*	Rhinolophidae	Caves	Malawi	Viphya Plateau	Kock et al. (1998)
	*Rhinolophus eloquens*	Rhinolophidae	Caves	Kenya	Mt. Elgon, Kapsakwany	Ferris and Usinger (1939), Kock et al. (1998)
	*Rhinolophus eloquens*	Rhinolophidae	Caves	South Africa	Gauteng (Formerly Transvaal)	Zumpt (1966)
	*Rhinolophus eloquens*	Rhinolophidae	Caves	South Sudan	Equatoria	Maa (1964)
	*Rhinolophus fumigatus*	Rhinolophidae	Caves	Malawi	Zomba	Kock et al. (1998)
	*Rhinolophus landeri*	Rhinolophidae	Caves	Democratic Republic of the Congo	Kasongo	Cooreman (1955)
	*Rhinolophus simulator*	Rhinolophidae	Caves	South Africa	Matlapitsi cave, GaMafefe, Linpopo Province	This study
	*Rhinolophus* sp.	Rhinolophidae	Caves	South Sudan	Torit	Maa (1964)
	*Rhinolophus* sp.	Rhinolophidae	Caves	Kenya	N Mambale	James D. Hawkins, Unpublished record (California Academy of Sciences, CA, USA)
	unknown	–	–	Somalia	Upper Sheika	Jordan (1912)
*Eoctenes coleurae* Maa (1964)	*Coleura afra*	Emballonuridae	Underground sites including caves	Sudan		Maa (1964)
*Eoctenes intermedius* Speiser (1904)	*Coleura afra*	Emballonuridae	Underground sites including caves	Guinea		Aellen (1956)
	*Taphozous mauritianus*	Emballonuridae	Mixed (no caves mentioned)	Democratic Republic of the Congo		Cooreman (1951)
	*Taphozous perforatus*	Emballonuridae	Underground sites including caves	Democratic Republic of the Congo	grotte Dethioux (Kataga)	Anciaux de Faveaux (1965), Benoit (1958); Leleup (1956)
	*Taphozous perforatus*	Emballonuridae	Underground sites including caves	Egypt	Luxor, Abu Rawash, Cairo	Maa (1961, 1964), Speiser (1904)
	*unknown host*	–	–	Sudan		Jordan (1912), Kellogg & Paine (1911)
*Eoctenes nycteridis* (Maa, 1964) and references therein)	*Nycteris arge*	Nycteridae	Mixed (no caves mentioned)	Liberia		Ferris and Usinger (1939)
	*Nycteris grandis*	Nycteridae	Mixed (no caves mentioned)	Congo		Cooreman (1951)
	*Nycteris hispida*	Nycteridae	Underground sites including caves	Tanzania	Victoria Nyanza, Shirati	Maa (1964) and references therein)
	*Nycteris hispida*	Nycteridae	Underground sites including caves	Rwanda		Benoit (1958)
	*Nycteris macrotis*	Nycteridae	Underground sites including caves	Democratic Republic of the Congo	Katanga	Anciaux de Faveaux (1965), Benoit (1958), Maa (1964)
	*Nycteris macrotis*	Nycteridae	Mixed but also caves	Rwanda		Benoit (1958)
	*Nycteris thebaica*	Nycteridae		Democratic Republic of the Congo	Katanga	Anciaux de Faveaux (1965)
	*Unknown host*	–	–	Eritrea	Sembel	Maa (1961, 1964)
	*Unknown host*	–	–	Uganda		Jordan (1912), Maa (1964)
*Hypoctenes clarus* (Jordan, 1922)	*Chaerephon pumilus* (currently *Mops pumilus*)	Molossidae	Mixed (no caves mentioned)	Congo		Benoit (1958)
	*Mops thersites*	Molossidae	Mixed (no caves mentioned)	Cameroon		Jordan (1922)
	*Mops thersites*	Molossidae	Mixed (no caves mentioned)	Ghana	Eastern Region	Maa (1970)
	*Otomops harrisoni*	Molossidae	Caves	Kenya		Patterson et al. (2018)
*Hypoctenes faini* Benoit (1958)	*Chaerephon pumilus* (currently *Mops pumilus*)	Molossidae	Mixed (no caves mentioned)	Kenya	Lake Naivasha	Greenwood (1991)
	*Otomops martiensseni*	Molossidae	Underground sites including caves	Rwanda	Ruhengeri	This study
	*Tadarida fulminans*	Molossidae	Underground sites including caves	Rwanda		Benoit (1958)

### 3.2 Geographical distribution of African polyctenids

**FIGURE 1:**
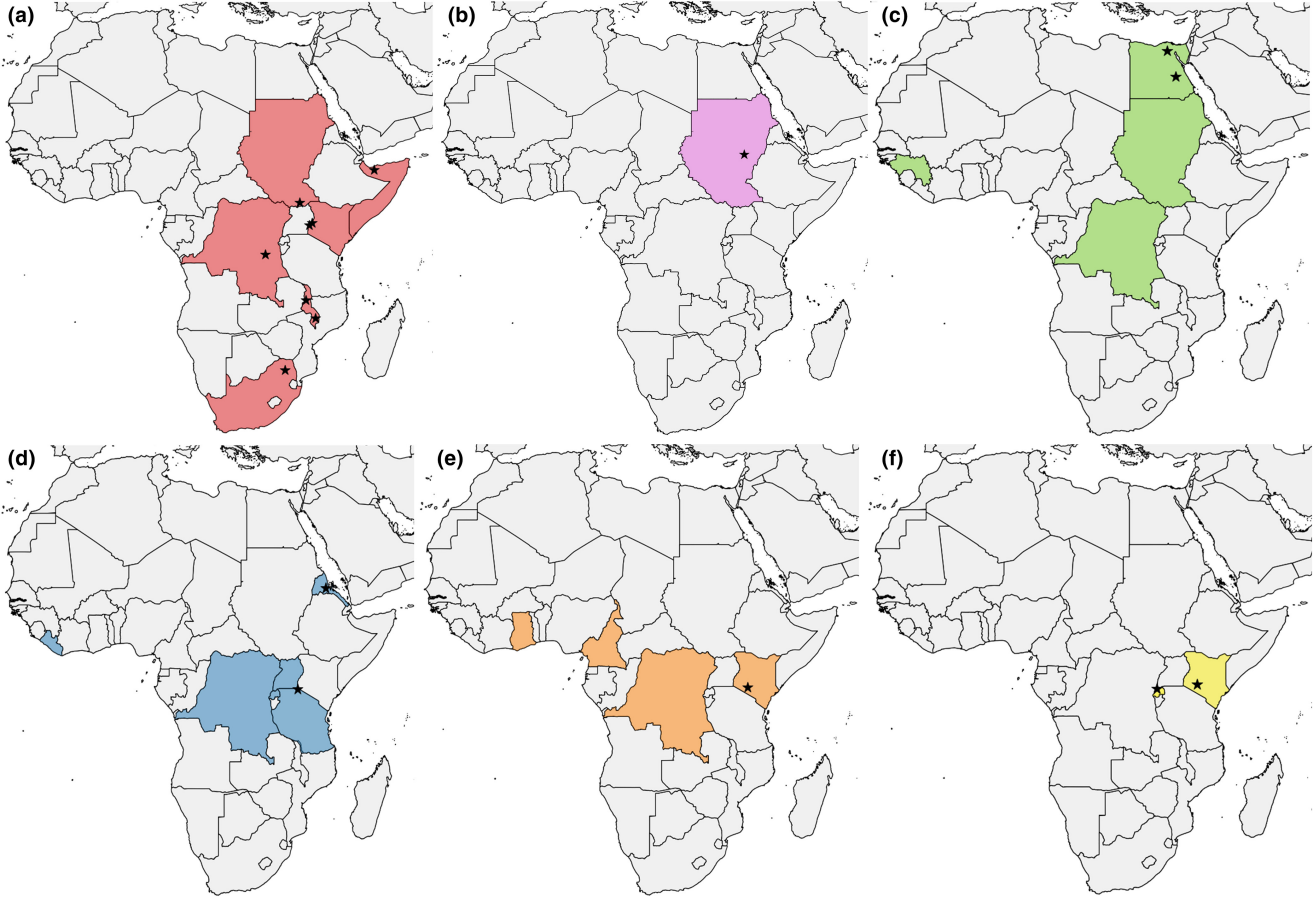
Distribution of Polyctenidae species in the African countries. Collection sites (whenever known) are indicated with black stars. *Adroctenes horvathi* (a), *Eoctenes coleurae* (b), *E. intermedius* (c), *E. nycteridis* (d), *Hypoctenes clarus* (e), and *H. faini* (f).

We collected distributional data of all six African polyctenid species, which have been reported from 14 countries to date (Figure 1a–f, Table 1). Our records of *A. horvathi* and *H. faini* are the second published occurrence of these species to both Rwanda and South Africa. Finally, *H. faini* and *A. horvathi* are reported for the first time from *Otomops martiensseni* and *Rhinolophus simulator*, respectively. We excluded records with unspecified data, when exact country was not given (e.g. “Central Africa”).

### 3.3 Infection patterns and sex ratio in Polyctenidae

Published and new records of Polyctenidae prevalence are shown in Table 2, including Old and New World species. Altogether, records of at least 2175 screened host individuals and 1716 parasites were obtained covering broad geographic scale. Most frequently, recorded prevalence rates are known from the New World genus *Hesperoctenes*. Sex ratio is often female biased in both New and Old World species; however, there is no clear evidence for strong female biased occurrence due to low sampling effort and lack of data. In total, 645 females and 381 males were reported from previous works, indicating female biased sex ratio (Table 2).

**TABLE 2:** Literature records and records of the present work indicating the infection patterns (prevalence), number of parasites (including sex and/or life stage), sex ratio, and location of the study

Parasite species	Host species	Hosts screened	Infected hosts (n)	Parasites (n)	Prevalence (%)	Female (n)	Male (n)	Nymph (n)	Biased sex ratio	Location	References
***Adroctenes horvathi***	*Rhinolophus* spp. (+unknown host species)	–	–	19	–	13	2	4	Female	Africa (various countries)	Maa (1964) and references therein)
	*Rhinolophus simulator*	41	1	1	2.4	1	0	0	–	South Africa	This study
***Eoctenes coleurae***	*Coleura afra*	–	–	4	–	2	1	1	–	Sudan	Maa (1964)
***Eoctenes intermedius***	*Taphozous* spp.	–	–	44	–	25	13	6	Female	Australia, Asia, Africa	Maa (1964) and references therein)
***Eoctenes nycteridis***	*Nycteris* spp.	–	–	26	–	14	1	11	Female	Africa (various countries)	Maa (1964) and references therein)
*Eoctenes spasmae*	*Megaderma spasma*	27	23	370	85.2	241	129	–	Female	Malaysia	Marshall (1982a)
	*Megaderma spasma* (+unknown)			102		51	27	24	Female	Asia (various countries)	Maa (1964) and references therein)
	*Megaderma spasma*	–	–	12	–	7	5	0	–	Philippines	Amarga & Yap (2017)
*Hesperoctenes angustatus*	*Molossus molossus*	20	1	1	5	–	–	–	–	Peru	Bonifaz et al. (2020)
*Hesperoctenes cartus*	*Cynomops planirostris* and *C. abrasus*	–	13	26	–	13	13	–	No	Argentina	Autino et al. (2020)
*Hesperoctenes fumarius*	Molossidae/emballonuridae/mormoopidae	–	–	148	–	45	53	50	No	South‐America (various countries)	Ueshima (1972)
*Hesperoctenes fumarius*	*Molossus rufus*	762	161	387	21	–	–	–	–	Brazil	Esbérard et al. (2005)
	*Molossus molossus*	228	70	–	26.8	–	–	–	–	Paraguay	Presley (2011)
	*Molossus rufus*	100	27	–	13	–	–	–	–	Paraguay	Presley (2011)
	*Molossus molossus*	228	62	106	27.1	31	28	47	No	Paraguay	Presley (2012)
	*Molossus bondae*	–	71	3	–	–	–	–	–	Columbia	Marinkelle & Grose (1979)
	*Molossus molossus*	3	–	6	–	6	0	–	–	Lesser Antilles	Smit & Miller (2019)
*Hesperoctenes longiceps*	*Eumops patagonicus*	526	89	135	16.9	52	33	50	Female	Paraguay	Presley (2012)
*Hesperoctenes parvulus*	*Molossops temminckii*	160	30	41	18.7	11	10	20	No	Paraguay	Presley (2012)
*Hesperoctenes vicinus*	*Molossops temminckii*	–	1	1	–	1	0	–	–	Argentina	Autino et al. (2020)
*Hesperoctenes* sp.	*Eumops glaucinus*	56	24	136	42.8	54	35	47	Female	Paraguay	Presley (2012)
	*Molossus molossus*	3	1	3	33	–	–	–	–	Colombia	Calonge‐Camargo & Pérez‐Torres (2018)
*Hesperoctenes* spp.	Molossidae/emballonuridae/mormoopidae	–	–	84	–	46	14	24	Female	South‐America (various countries)	Ueshima (1972)
***Hypoctenes clarus***	*Otomops harrisoni*	20	5	5	25	2	3	0	–	Kenya	Patterson et al. (2018)
	*Tadarida* spp.	–	–	3	–	2	1	0	–	Africa (various countries)	Maa (1964) and references therein)
	*Tadarida thersites* (currently *Mops thersites*)	–	–	12	–	8	1	3	Female	Ghana	Maa (1970)
***Hypoctenes faini***	*Tadarida fulminans*	–	–	1	–	1	0	0	–	Rwanda	Maa (1964) and references therein)
	*Otomops martiensseni*	1	1	1	(100)	1	0	0	–	Rwanda	This study
*Hypoctenes hutsoni*	*Tadarida pusilla (currently Mops pusillus*) (+unknown)	–	–	23	–	10	10	3	No	Seychelles	Maa (1970)
*Polyctenis molossus*	*Megaderma lyra* (currently *Lyroderma lyra*) (+unkown)	–	–	16	–	8	2	6	Female	Asia (various countries)	Maa (1964) and references therein)

*Note*: African species are highlighted in bold.

### 3.4 Molecular analysis of COI, 16S, 18S, and 28S rRNA gene

Based on BLAST search, for the COI gene fragment the closest match for *H. fainii* and *A. horvathi* was 83.09% *Psacasta exanthematica* (MF162983) (Scutelleridae) and 83.18% Ceratocapsidea (MW984087), respectively. The 16S sequences of *H. fainii* and *A. horvathi* showed the highest similarity of 84.29% *Tetraphleps aterrimus* (NC_042679) (Anthocoridae) and 83.65% *Primicimex cavernis* (MG596876) (Cimicidae), respectively. For the 18S fragment, *H. fainii* and *A. horvathi* showed the highest similarity to 97.37% and 95.50% *Latrocimex spectans* (MZ378786) (Cimicidae), respectively. Lastly, the BLAST search of the 28S gene fragment of *A. horvathi* (28S) showed a 90.12% similarity with *Cimex lectularius* (KJ461188) (Cimicidae). Amplification and sequencing of the 28S rRNA gene of *H. faini* were unsuccessful with two different primer sets.

**FIGURE 2:**
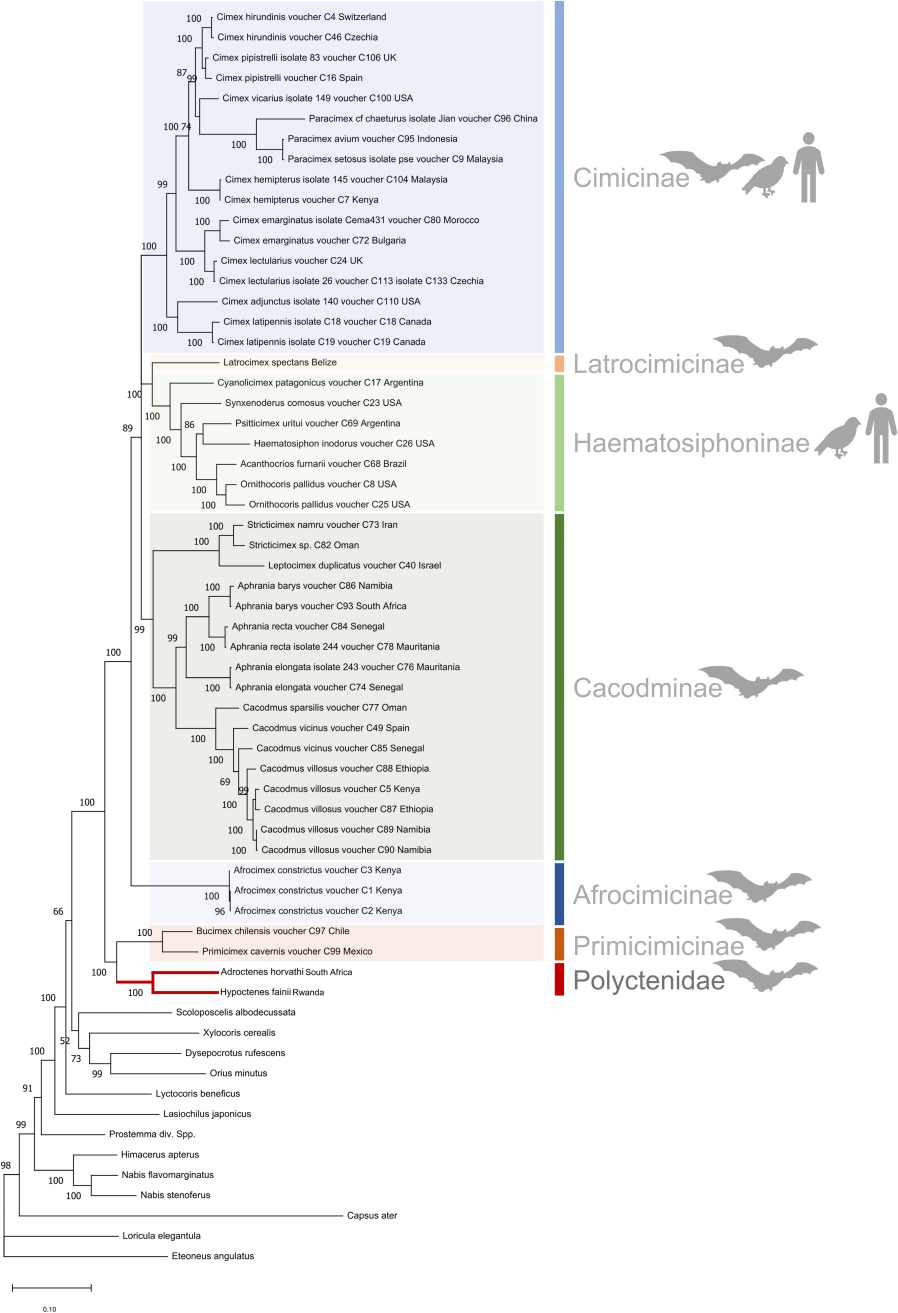
Bayesian tree of family Cimicidae (including all six subfamilies) and Polyctenidae (including two species, one subfamily) based on concatenated sequences of the cytochrome c oxidase subunit 1 (COI), 16S, and 18S rRNA genes. GenBank accession numbers for each species are indicated in Table 3. Scale bar indicates the number of substitutions per site. Main host groups are indicated for each subfamily (i.e., birds, bats, and and/or humans).

Overall, within Cimicoidea, as reflected by the topology of the Bayesian tree based on three genetic markers (COI, 16S, and 18S rRNA genes) (Figure 2, Table 3), the monophyly of Cimicidae can only be maintained if it includes Polyctenidae.

**TABLE 3:** Molecular data used in this study to show phylogenetic relationship between Cimicidae and Polyctenidae, with GenBank accession numbers (COI, 16S, and 18S)

Species	Host group	Host species	Host family	Country	COI	16S	18S
*Acanthocrios furnarii*	Bird	*Furnarius rufus* (nest)	Furnariidae	Brazil	MG596830	MG596866	MG978385
*Adroctenes horvathi*	Bat	*Rhinolophus simulator*	Rhinolophidae	South Africa	This study	This study	This study
*Afrocimex constrictus*	Bat	*Rousettus aegyptiacus*	Pteropodidae	Kenya	MG596804	MG596841	MG978357
*Afrocimex constrictus*	Bat	*Rousettus aegyptiacus*	Pteropodidae	Kenya	MG596805	MG596842	MG978358
*Afrocimex constrictus*	Bat	*Rousettus aegyptiacus*	Pteropodidae	Kenya	MG596806	MG596843	MG978359
*Aphrania barys*	Bat	*Neoromicia capensis* (currently *Laephotis capensis*)	Vespertilionidae	Namibia	MG596820	MG596856	MG978375
*Aphrania barys*	Bat	*Neoromicia capensis* (currently *Laephotis capensis*)	Vespertilionidae	South Africa	MG596825	MG596861	MG978380
*Aphrania elongata*	–	Unknown	–	Senegal	MG596812	MG596849	MG978367
*Aphrania elongata*	Bat	*Scotophilus leucogaster*	Vespertilionidae	Mauritania	KF018763	KF018729	KF018715
*Aphrania recta*	Bat	*Nycticeinops schlieffeni*	Vespertilionidae	Mauritania	KF018764	KF018730	KF018716
*Aphrania recta*	Bat	*Neoromicia* cf. *guineensis*	Vespertilionidae	Senegal	MG596818	MG596854	MG978373
*Bucimex chilensis*	–	Unknown	–	Chile	MG596840	MG596877	MG978399
*Cacodmus sparsilis*	Bat	*Pipistrellus dhofarensis*	Vespertilionidae	Oman	MG596813	MG596850	MG978369
*Cacodmus vicinus*	Bat	*Pipistrellus* sp.	Vespertilionidae	Spain	MG596816	MG596852	MG978371
*Cacodmus vicinus*	Bat	*Scotoecus hirundo*	Vespertilionidae	Senegal	MG596819	MG596855	MG978374
*Cacodmus villosus*	–	Unknown	–	Kenya	MG596815	MG596851	MG978370
*Cacodmus villosus*	Bat	*Pipistrellus hesperidus*	Vespertilionidae	Ethiophia	MG596821	MG596857	MG978376
*Cacodmus villosus*	Bat	*Pipistrellus hesperidus*	Vespertilionidae	Ethiophia	MG596822	MG596858	MG978377
*Cacodmus villosus*	Bat	*Neoromicia capensis* (currently *Laephotis capensis*)	Vespertilionidae	Namibia	MG596823	MG596859	MG978378
*Cacodmus villosus*	Bat	*Neoromicia capensis* (currently *Laephotis capensis*)	Vespertilionidae	Namibia	MG596824	MG596860	MG978379
*Cimex adjunctus*	Bat	*Nycticeius humeralis*	Vespertilionidae	USA	GU985536	GU985558	KF018712
*Cimex emarginatus*	Bat	*Myotis* cf. *alcathoe*	Vespertilionidae	Bulgaria	MG596837	MG596874	MG978396
*Cimex emarginatus*	Bat	*Pipistrellus pipistrellus*	Vespertilionidae	Morocco	MF680526	MF680517	MG978397
*Cimex hemipterus*	Human	*Homo sapiens*	Hominidae	Kenya	MG596826	MG596862	MG978381
*Cimex hemipterus*	Human	*Homo sapiens*	Hominidae	Malaysia	KF018754	KF018724	KF018710
*Cimex hemipterus*	Human	*Homo sapiens*	Hominidae	India	KF018755	KF018725	KF018710
*Cimex hirundinis*	–	Unknown	–	Switzerland	MG596808	MG596845	MG978363
*Cimex hirundinis*	Bird	*Delichon urbica*	Hirundinidae	Czechia	MG596809	MG596846	MG978364
*Cimex latipennis*	Bat	*Myotis lucifugus*	Vespertilionidae	Canada	KF018758	KF018734	KF018720
*Cimex latipennis*	Bat	*Myotis volans*	Vespertilionidae	Canada	KF018757	KF018733	KF018719
*Cimex lectularius*	Human	*Homo sapiens*	Hominidae	Czechia	GU985524	GU985546	KF018711
*Cimex lectularius*	Human	*Homo sapiens*	Hominidae	UK	MG596836	MG596873	MG978394
*Cimex pipistrelli*	Bat	*Pipistrellus* sp.	Vespertilionidae	UK	GU985534	GU985556	MG978393
*Cimex pipistrelli*	Bat	Chiroptera	–	Spain	MG596835	MG596872	MG978392
*Cimex vicarius*	Bird	*Petrochelidon pyrrhonota*	Hirundinidae	USA	GU985541	GU985563	KF018709
*Cyanolicimex patagonicus*	Bird	*Cyanoliseus patagonus*	Psittacidae	Argentina	MG596833	MG596869	MG978388
*Haematosiphon inodorus*	Bird	*Falco mexicanus* (nest)	Falconidae	USA	MG596829	MG596865	MG978384
*Hypoctenes faini*	Bat	*Otomops martiensseni*	Molossidae	Rwanda	This study	This study	This study
*Latrocimex spectans*	Bat	*Noctilio leporinus*	Noctilionidae	Belize	MW269881	MW270938	MZ378786
*Leptocimex duplicatus*	–	Unknown	–	Israel	MG596810	MG596847	MG978365
*Ornithocoris pallidus*	–	Unknown	–	USA	MG596827	MG596863	MG978382
*Ornithocoris pallidus*	Bird	*Delichon urbicum* (nest)	Hirundinidae	USA	MG596828	MG596864	MG978383
*Paracimex avium*	Bird	*Aerodramus salanganus*	Apodidae	Indonesia	MG596807	MG596844	MG978360
*Paracimex* cf *chaeturus*	Bird	*Aerodramus brevirostris*	Apodidae	China	MF680531	MF680520	MG978362
*Paracimex setosus*	Bird	*Aerodromus* sp.	Apodidae	Malaysia	KF018761	KF018735	KF018721
*Primicimex cavernis*	Bat	*Tadarida brasiliensis*	Molossidae	Mexico	MG596839	MG596876	MG978398
*Psitticimex uritui*	Bird	*Myiopsitta monachus*	Psittacidae	Argentina	MG596831	MG596867	MG978386
*Stricticimex namru*	Bat	mixed species bat colony	–	Iran	MG596811	MG596848	MG978366
*Stricticimex* sp.	Bat	*Nyctinomus thomasi* (currently *Tadarida aegyptiaca*)	Molossidae	Oman	MG596817	MG596853	MG978372
*Synxenoderus comosus*	Bird	*Aeronautes saxatalis* (nest)	Apodidae	USA	MG596832	MG596868	MG978387
*Amphiareus obscuriceps*	Outgroup		Anthocoridae		GQ292178	GQ258358	GQ258393
*Anthocoris confusus*	Outgroup		Anthocoridae		KM022525	GQ258359	GQ258401
*Blaptostethus aurivillus*	Outgroup		Anthocoridae		KF36463	GQ258388	GQ258400
*Buchananiella crassicornis*	Outgroup		Anthocoridae		GQ292145	GQ258364	GQ258407
*Capsus ater*	Outgroup		Miridae		AY252977	AY252712	EU683117
*Dysepicritus rufescens*	Outgroup		Anthocoridae		GQ292210	GQ258386	GQ258399
*Eteoneus angulatus*	Outgroup		Tingidae		EF523481	EF487290	EF487311
*Himacerus apterus*	Outgroup		Nabidae		KR034788	GQ258381	GQ258425
*Lasiochilus japonicus*	Outgroup		Anthocoridae		GQ292187	GQ258367	GQ258410
*Loricula elegantula*	Outgroup		Microphysidae		KM022867	EU683098	EU683151
*Lyctocoris beneficus*	Outgroup		Lyctocoridae		GQ292284	GQ258369	GQ258412
*Nabis flavomarginatus*	Outgroup		Nabidae		KM022694	GQ258380	GQ258424
*Nabis stenoferus*	Outgroup		Nabidae		GQ292211	GQ258379	GQ258426
*Orius minutus*	Outgroup		Anthocoridae		KR040183	GQ258372	GQ258417
*Prostemma div*. spp.	Outgroup		Nabidae		JQ782833	JQ782833	JQ782787
*Scoloposcelis albodecussata*	Outgroup		Anthocoridae		GQ292129	GQ258376	GQ258422
*Xylocoris cerealis*	Outgroup		Anthocoridae		GQ292172	GQ258384	GQ258395

## 4 DISCUSSION

### 4.1 Distribution of polyctenidae in Africa

Currently, six species of polyctenids are known from the African region. *Adroctenes horvathi* has been recorded in the African continent only and has the widest distribution, being present in Eastern and Southern Africa and is the most common species among all the known African polyctenids. The primary host species of *A. horvathi* belong to the family Rhinolophidae, which are widely distributed in continental Africa and *A. horvathi* may be present in additional countries where its presence has not yet been observed.

*Eoctenes* is the most species‐rich genus in Africa, with three different species. Nevertheless, *E. coleurae* seems to be the most rarely collected polyctenid species among all the African Polyctenidae as it has been recorded only once in Sudan and has not been reported since its description (Maa, 1964), making additional conclusions on its distribution problematic. Nevertheless, its host *Coleura afra* is a widely distributed species, known from several Central, Eastern, and Western African countries. Consequently, *E. coleurae* might occur within its host distribution (if *C. afra* is the main host of this species). Future studies focusing on family Emballonuridae and its parasitic fauna should give more insights to the distribution of *E. coleurae*.

*Eoctenes nycteridis* is also endemic to the African continent and has been mostly reported from the central countries with some additional records, such as Eritrea and Liberia; therefore, it is expected to occur in other regions within the distribution range of its hosts, family Nycteridae. Species belonging to family Nycteridae occur in Africa but some parts of Asia as well.

*Eoctenes intermedius* is a widely distributed species with several records from Asia (Maa, 1961, 1964, Theodor & Moscona, 1954), Australia (Maa, 1964), and Africa (Jordan, 1912; Maa, 1964; Speiser, 1904). In Africa, the species has a Northern and Central African distribution but has also been recorded once in Guinea, Western Africa (Aellen, 1956). Its hosts, *C. afra* and *Taphozous* spp., are widely distributed in Africa, *T. perforatus* occurring in several parts of Asia as well. Within its global distribution, *E. intermedius* shows a strong preference toward *Taphozous* species; therefore, its distribution is expected where these hosts occur (Maa, 1964).

The genus *Hypoctenes* includes two species, *H. clarus* and *H. faini* exclusively found in the African continent. The African representatives of this genus are rarely collected, and records seem to be limited in a relatively narrow distribution, when compared to other species in the family. *Hypoctenes clarus* has been reported from Cameroon, Democratic Republic of Congo, Ghana, and Kenya (Benoit, 1958; Jordan, 1922; Maa, 1970; Patterson et al., 2018). It might have additional populations in other regions where host species are distributed. Family Molossidae is one of the most species rich bat families occurring in all continents (except Antarctica) (Ammerman et al., 2012). *Hypoctenes clarus* and *H. faini* are known to occur on the members of this family but reports are scarce.

*Hypoctenes faini* is also a rarely observed species, with only two published records, representing two specimens (Benoit, 1958; Greenwood, 1991). During our work, two specimens of *H. faini* have been found in Rwanda for the second time (Figure 1). It might be expected from additional countries where its potential hosts from the Molossidae family are present. *Otomops martiensseni*, which we recorded in Rwanda as host species, occurs mainly in Central Africa but has populations in the southern and western part of the continent; therefore, the occurrence of *H. faini* is possible in these areas.

### 4.2 Host specificity

Based on literature and field collected data, all polyctenid species appeared to be oligoxenous, meaning that they occur on two or more congeneric host species. However, the number of sampled individuals is low and conclusions cannot be drawn on the preferred host species, if any. Nevertheless, all polyctenid species exclusively occur on the members of a single bat family. The level of dispersal ability of polyctenids is unknown, although Marshall (1981) stated that biased sex ratio occurs in polyctenids due to males being the more mobile sex (Marshall, 1981), which could affect their dispersal ability and their specificity. Phylogenetic specificity (rather than ecological specificity) is supported by the fact that some host species often form mixed colonies with bats belonging to different families, which are not known as polyctenid hosts (McDonald et al., 1990; van der Merwe 1987). In conclusion, dispersal barriers do not likely influence polyctenid host specificity.

Common characteristics of polyctenid hosts include insectivore behavior; however, emballonurids occasionally consume fruits. Infected bat species mostly roost in underground places, such as caves. The microclimate of these roosts might be preferred or required by polyctenids.

### 4.3 Sex ratio and infection patterns

Biased sex ratio in ectoparasitic insects is common and has been explored in the case of bat‐associated parasites (Dick & Patterson, 2008; Dittmar et al., 2011; Szentiványi et al., 2017). Several factors may cause biased sex ratio, such as difference between body size, mobility, dispersal ability between sexes, or the presence of reproduction manipulating bacteria or inbreeding (Dick & Patterson, 2008; Duron et al., 2008; Patterson et al., 2008; Szentiványi et al., 2017). We found some evidence of female biased sex ratio in polyctenid bat bugs, similarly to previous suggestion (Marshall, 1982a). Overall, it is currently unknown if polyctenid bat bugs show biased sex ratio at birth, such as in the case of bat flies (Dittmar et al., 2011), or if female biased sex ratio occurs a later life stage. If natural polyctenid populations are truly female biased, some scenarios (or combinations of them) might explain this phenomenon. Local mate competition (LMC) could be one explanation. LMC results a female biased sex ratio in parasite populations, due to dispersal limited, isolated, and inbred populations, which could all be true in the case of polyctenids. LMC implies a female biased sex ratio, since males compete for mating opportunities, and mothers try to decrease sexual competition by maximizing female success through reducing the number of male offspring (Hamilton, 1967). Marshall (1982a, 1982b) suggested that biased sex ratio occurs because males are more active than females and therefore more exposed to predation by their hosts (Marshall, 1981, 1982a). Additionally, if there is different mobility and dispersal ability between sexes, it might also affect the capture success and thus implies a apparent bias in sex ratio. Furthermore, different longevity between females and males might also strongly influence sex ratio. Dispersal ability and mobility differences between female and male polyctenids are currently unknown on their hosts; however, off‐host both sexes are incapable of moving (Marshall, 1982a). Additionally, *Wolbachia*, which is a genus of Gram‐negative bacteria known to be able to alter sex ratios, has been found at least in one polyctenid species, *Hesperoctenes fumarius* (Sakamoto et al., 2006), and is common in other bat ectoparasites (Morse et al., 2012; Wilkinson et al., 2016). Nevertheless, there is a lack of evidence if they occur in a wide range of polyctenid species, and if they affect their reproduction. Future studies should address polyctenid sex ratios and their driving factors.

Prevalence of polyctenids shows a wide variation on their hosts, ranging from 2.4% to 85.2%. We currently have little understanding on what affects prevalence of these ectoparasites, although it is likely shaped by several factors, such as host availability, dispersal ability, seasonality, and population dynamics of each species. Furthermore, data on potential host sex bias are not available or scarce; however, one study found equal infection between female and male hosts (Marshall, 1982b). Prevalence and infection pattern between host sexes need to be explored in future studies.

### 4.4 Phylogenetic relationship of Polyctenidae

Previous phylogenetic trees involving Polyctenidae were based on morphological data (Schuh et al., 2009). Our genetic analysis placed Primicimicinae (*Primicimex* and *Bucimex*) to the base of the tree. Polyctenid species cluster close to Primicimicinae, forming a separate clade at the base of Cimicidae. Based on these results, Polycteninae is a sister clade to Primicimicinae. Subfamily Cacodminae appears to be monophyletic, which has been shown before (Balvín et al., 2015; Hornok et al., 2021; Ossa et al., 2019; Roth et al., 2019). Subfamily Cimicinae also shows monophyly, with two separated clusters for the genus *Cimex* encompassing the genus *Paracimex*, which supports previous findings (Balvín et al., 2015; Roth et al., 2019). Furthermore, the 18S sequence of *H. faini* was only 93.5% identical to *Curalium cronini* (EU683128) suggesting that Curaliidae is not a sister group of Polyctenidae (unlike in Schuh et al. (2009): figure 10). Until today, there is a single 18S sequence available for *Curalium cronini*, representing family Curaliidae, and further conclusion cannot be drawn regarding its relationship to the Polyctenidae family.

Based on previous works, we expected Polyctenidae and Cimicidae to be two separate monophyletic group on their own; our results strongly suggest that the monophyly of Cimicidae can only be maintained if it includes Polyctenidae. However future studies including more polyctenid species are needed to draw final conclusions. Overall, family Polyctenidae (or subfamily Polycteninae) may be considered as a subfamily of Cimicidae.

### 4.5 Potential as vectors

Polyctenidae have not been identified as vectors of any pathogens. However, they may have a potential role in disease transmission. Closely related bat bug species belonging to family Cimicidae are competent or suspected vectors of several pathogens, such as *Trypanosoma*, *Bartonella*, and Kaeng Khoi virus (Gardner & Molyneux, 1988; Reeves et al., 2005; Salazar et al., 2015; Van Den Berghe et al., 1963; Williams et al., 1976). The vector of *Nycteria* (Haemosporidia) parasites, which have been shown to infect, e.g., Rhinolophidae and Nycteridae species (Schaer et al., 2015), is not known and as some polyctenids parasitize these families, it is possible that they play a vectorial role in *Nycteria* transmission.

## AUTHOR CONTRIBUTIONS

**Tamara Szentiványi:** Conceptualization (equal); data curation (equal); formal analysis (equal); investigation (equal); methodology (equal); project administration (equal); resources (equal); supervision (equal); visualization (equal); writing – original draft (equal); writing – review and editing (equal). **Sándor Hornok:** Conceptualization (equal); data curation (equal); methodology (equal); supervision (equal); writing – original draft (equal); writing – review and editing (equal). **Áron Botond Kovács:** Formal analysis (equal); methodology (equal); writing – review and editing (equal). **Nóra Takács:** Methodology (equal); writing – review and editing (equal). **Miklós Gyuranecz:** Formal analysis (equal); software (equal); writing – review and editing (equal). **Wanda Markotter:** Data curation (equal); funding acquisition (equal); investigation (equal); supervision (equal); writing – review and editing (equal). **Philippe Christe:** Conceptualization (equal); project administration (equal); supervision (equal); validation (equal); writing – original draft (equal); writing – review and editing (equal). **Olivier Glaizot:** Conceptualization (equal); project administration (equal); supervision (equal); validation (equal); writing – original draft (equal); writing – review and editing (equal).

## CONFLICT OF INTEREST

The authors declare no competing interests.

## ETHICS APPROVAL

Ethical approval was obtained from the University of Pretoria (Pretoria, South Africa; EC054–14) and Research was performed under Section 20 approval of the Department of Agriculture, Land Reform and Rural Development, South Africa.

## Data Availability

The data that support the findings of this study are openly available in GenBank at ncbi.nlm.nih.gov/genbank/, reference number ON157489 ‐ ON182061.
